# Risk of first hip fracture under treatment with zoledronic acid versus alendronate: a NOREPOS cohort study of 88,000 Norwegian men and women in outpatient care

**DOI:** 10.1007/s11657-024-01458-4

**Published:** 2024-10-23

**Authors:** Brit Solvor Lyse Riska, Nina Gunnes, Trine E. Finnes, Haakon E. Meyer, Mari Hoff, Tone K. Omsland, Kristin Holvik

**Affiliations:** 1 Norwegian Research Centre for Women’s Health, Oslo University Hospital, Oslo, Norway; 2 Department of Physical Health and Ageing, Norwegian Institute of Public Health, Oslo, Norway; 3 Department of Community Medicine and Global Health, University of Oslo, Oslo, Norway; 4 Department of Endocrinology, Innlandet Hospital Trust, Hamar, Norway; 5 Department of Endocrinology, Oslo University Hospital, Oslo, Norway; 6 Department of Neuromedicine and Movement Science, NTNU Norwegian University of Science and Technology, Trondheim, Norway; 7 Department of Rheumatology, St. Olavs Hospital, Trondheim, Norway

**Keywords:** Osteoporosis, Hip fracture, Bisphosphonate, Alendronate, Zoledronic acid, Real-world data

## Abstract

**Summary:**

We aimed to investigate the risk of hip fracture associated with zoledronic acid treatment compared to alendronate on a population level. The risk of hip fracture was lower in women using zoledronic acid and higher in women who had discontinued treatment. The findings support the effectiveness of intravenous bisphosphonate.

**Purpose:**

To investigate whether zoledronic acid (ZOL) was associated with a lower risk of the first hip fracture than alendronate (ALN) in Norway using real-world data.

**Methods:**

Nationwide data on drugs dispensed in outpatient pharmacies were individually linked with all hospital-treated hip fractures. Individuals aged 50–89 years without previous hip fracture were included at their first filling of a prescription for ALN or ZOL during 2005–2016. Hazard ratios (HRs) with 95% confidence intervals (95% CIs) for first hip fracture by time-varying exposure to ZOL versus ALN were estimated in sex-stratified flexible parametric survival analyses. Covariates included time-varying accumulated ALN exposure and comorbidity level expressed by the prescription-based Rx-Risk Comorbidity Index, marital status, education, and residential urbanity.

**Results:**

Of 75,250 women who initiated treatment, 72,614 (96.5%) were exposed to ALN and 6366 (8.5%) to ZOL. Of 12,739 men who initiated treatment, 12,311 (96.6%) were exposed to ALN and 784 (6.2%) to ZOL. In women, the HR for first hip fracture was 0.75 (95% CI: 0.61–0.91) for ZOL versus ALN. In men, the corresponding HR was 0.59 (95% CI: 0.32–1.07). Discontinued treatment was associated with increased risk compared with current ALN treatment in women (HR: 1.33; 95% CI: 1.24–1.42, men: HR 1.13 (95% CI: 0.95–1.35)).

**Conclusions:**

In women, the risk of first hip fracture when treated with ZOL was 25% lower than when treated with ALN. Discontinued treatment was associated with a 33% increase in hip fracture risk. Similar, albeit statistically non-significant, results were observed in men.

## Introduction

Fragility fractures will be an increasingly important clinical and societal challenge as the proportion of older people in the population will increase dramatically during the next few decades [1, 2]. Among the fragility fractures, hip fractures cause the largest excess mortality and require the most resources within the healthcare services [3]. Norway has among the world’s highest incidence rates of hip fracture [4]. Drugs that affect bone metabolism and prevent bone loss have a large fracture-preventive potential in high-risk individuals [5]. Alendronate (ALN), a bisphosphonate usually administered as weekly oral tablets, is the most commonly prescribed anti-osteoporosis drug [6]. Zoledronic acid (ZOL), a bisphosphonate administered as a yearly intravenous infusion, is a more recent alternative, and its use has increased since its introduction in Norway in 2005 [6, 7]. In clinical trials of postmenopausal women with osteoporosis, ALN and ZOL showed relative risks (RRs) of hip fracture of 0.47 and 0.59, respectively, compared to placebo after 3–4 years of treatment [8, 9]. A recent meta-analysis of randomized controlled trials showed a 35% reduced risk of hip fracture after 24 months of treatment with a bisphosphonate compared to placebo in osteoporotic postmenopausal women [10]. Both medications have shown similar estimates of time-to-onset of antifracture effect, with statistically significant protection from 12 months of treatment [8, 9, 11, 12]. A re-evaluation of indication and a possible treatment break (“drug holiday”) are recommended for both treatments after five (ALN) or three (ZOL) years [13].

Few head-to-head studies have compared the effectiveness of ALN and ZOL in hip fracture prevention, either in clinical trials or in observational studies using real-world data. One clinical trial compared the two drugs in terms of bone turnover, with ZOL having the most favorable outcome [14]. A meta-analysis of eight clinical trials concluded that ZOL appeared to offer the best overall fracture protection when compared to placebo [15]. A registry-based cohort study using Swedish and Dutch data, however, found no statistically significant difference in fracture risk between women treated with ZOL and women treated with ALN [16]. For men the data are even more scarce. Bisphosphonates seem to prevent fractures in osteoporotic men [17], and some studies indicate that there is no statistically significant difference in efficacy between ZOL and ALN [18, 19].

Oral regimens of preventive osteoporosis medications are susceptible to poor adherence and lack of persistence [20], both to which treatment effectiveness is intimately linked [21]. Efficacy of and adherence to oral ALN may also be affected by its instructions for administration, requiring a fasting state, an upright position, and no consumption of food or water for at least half an hour after ingestion [22]. Concurrent food intake markedly inhibits absorption and thus bioavailability [23]. An intravenous regimen with once-yearly administration of ZOL will not be associated with similar adherence problems [20].

With this backdrop, we aimed to investigate whether ALN and ZOL had similar associated hip fracture risk on a population level in Norway, a country with a particularly high hip fracture incidence.

## Methods

### Study population

We conducted a registry-based nationwide open cohort study with a prevalent new-user design, including new users of ZOL and ALN and allowing individuals to discontinue use, restart use, and switch between the two drugs [24]. The study population included all Norwegian women and men aged 50–89 years with no previous hip fracture (based on look-back to January 1, 1994) who initiated drug treatment with ALN or ZOL at any time from July 1, 2005, to December 31, 2016, based on the Norwegian Prescription Database (NorPD). Information on residential status and date of death or emigration was obtained from the National Population Register, provided by Statistics Norway.

### Exposure

Exposure data were based on filled prescriptions registered in the NorPD, where all drugs dispensed by pharmacies in Norway since 2004 are registered. The NorPD is based on mandatory electronic reports from pharmacies on dispensed medications [25]. Drugs administered in hospitals, including public outpatient clinics and nursing homes, are not included, nor are over-the-counter drugs. For each prescription filling, individual-level data on the fill date (monthly precision), drug name, dosage form, marketing name, and dispensed amount were available, as well as sex and year and month of birth of the patient. The drugs are classified according to the Anatomical Therapeutic Chemical (ATC) Classification System, and dispensed amount is registered in defined daily doses (DDDs), as per the World Health Organization Collaborating Centre for Drug Statistics Methodology [26]. The existing DDD of ZOL is based on the indication tumor-induced hypercalcemia [26], and for our purpose, the DDDs were adjusted to match the dosage in osteoporosis treatment as listed in the Norwegian Pharmaceutical Product Compendium [27]. Individuals were included in the month of their first filling of a prescription for either ALN or ZOL in an outpatient pharmacy in Norway following an 18-month washout period for any bisphosphonate (ATC M05BA with all subgroups) or denosumab (ATC M05BX04). ZOL for osteoporosis was distinguished from ZOL for other indications by its product name and dosage form of 5 mg/100 ml, and only this formulation was included. For both drug types, users were defined as exposed for the number of days corresponding to the amount dispensed (DDDs) with an addition of 6 months to account for the documented persisting effect of these drugs [28, 29]. Exposures to the respective study drugs were treated as two separate time-varying binary variables, assigned a value of 1 at first filling (inclusion) and changing to a value of 0 when the exposure period expired without renewed filling. At any later renewed filling the exposure level would again change to 1. Hence, each individual could at any point during follow-up have one of the following three statuses: exposed to ALN only, exposed to ZOL, and unexposed (i.e., discontinued treatment).

### Outcome

The outcome was first-time hip fracture. Data on all hip fractures treated in hospitals in Norway since 1994 were available in the Norwegian Epidemiologic Osteoporosis Studies (NOREPOS) hip fracture database. The data are quality assured, and admissions included are for incident hip fractures (http://www.norepos.no/documentation) [30].

### Covariates

Demographic covariates included sex, decade of birth (to account for cohort effects and secular changes in fracture risk), marital status (married/registered partner or other), educational level, and urbanization level of municipality of residence. These were all obtained from the Norwegian Population and Housing Census 2001. Educational level was grouped according to the Norwegian Standard Classification of Education and further combined into three levels: low (0–10 years of schooling), medium (11–14 years of schooling, including post-secondary schooling but not higher-level education), and high (undergraduate, graduate, and postgraduate education) [31]. Urbanization level of municipality of residence was based on the proportion of densely spaced housing (i.e., < 50 m between houses): low (rural: less than one-third), medium (semirural: between one-third and two-thirds), and high (urban: more than two-thirds) [32].

To account for variation in comorbidity, we applied the prescription-based ATC-mapped Rx-Risk Comorbidity Index [33]. Time-varying Rx-Risk scores were calculated for each individual based on filled prescriptions in the NorPD, updated every other calendar year. The score was constructed by summing up the severity weights for each chronic condition (out of 45 possible; osteoporosis not included) for which the individual filled a prescription in a specific calendar year. Each category has been assigned an empirical severity weight from -1 through 6 according to the age- and sex-adjusted one-year mortality associated with the category [33]. Rx-Risk scores were grouped into 13 categories (< 0, 0, 1, …, 9, ≥ 10, or missing). We used last observation carried forward imputation if an individual’s Rx-Risk score was missing for a specific two-year period.

### Statistical analysis

Hazard ratios (HRs) with 95% confidence intervals (CIs) for hip fracture during exposure to ZOL versus ALN (reference category) were estimated using flexible parametric survival models. Individuals were followed until first-time hip fracture (outcome of interest) or censoring due to death, emigration, month of 90^th^ birthday, filling of a prescription for other bisphosphonates (including etidronic acid, clodronic acid, pamidronic acid, tiludronic acid, risedronic acid, and ZOL 4 mg/100 ml) or denosumab, or end of study on December 31, 2016, whichever occurred first. Any individual who entered the cohort by filling a prescription could transition between three statuses until hip fracture or censoring, namely, current ALN exposure (denominator), current ZOL exposure, or no exposure (discontinued). Time intervals with concurrent exposure to both medications were grouped in the ZOL exposure category on the presumption that this will usually represent a time interval with leftover oral ALN tablets from a previous filling when receiving an infusion of ZOL.

All analyses were performed separately for women and men. A base model was fitted with age as timescale and no covariates, and two adjusted models were fitted: Model I included all covariates except accumulated exposure time to ALN, which was added in Model II. We used time-varying exposure status and Rx-Risk scores. First, models without time-dependent effects of exposure were fitted to yield overall HR estimates, assuming proportional hazards [34]. Second, we fitted a version of Model II allowing time-dependent effects of drug exposure, by including the *tvc* option in the *stpm2* command for flexible parametric survival analysis in Stata, estimating HR over time (age in months).

We performed an additional analysis stratified by treatment duration. HRs with 95% CIs were thus estimated within time intervals grouped by accumulated exposure for both drug types. Accumulated exposure was calculated for each individual for each drug, disregarding pauses in treatment, and was grouped in five categories: 1–5 months, 6–11 months, 12–23 months, 24–35 months, and ≥ 36 months. For each of the two drug types, an indicator variable for each length of accumulated treatment was constructed and assigned a value of 1 for current exposure to the drug and 0 otherwise. This analysis was carried out in women only due to the limited person-time of ZOL exposure and correspondingly few hip fractures among men.

In addition, two sensitivity analyses were performed in women, censoring patients in the month of reaching age 85 years and 80 years, respectively, to reduce the impact of potential misclassification introduced by nursing home admissions among the oldest. Sensitivity analyses were also performed with the inclusion of a categorical variable (0/1) for ever-use of each of the following: teriparatide (ATC H05AA02), glucocorticoids (GCs) (H02AB), sedatives and anxiolytics (ATC N05C and N05B) and opioids (ATC N02A).

All statistical analyses were performed using Stata/SE 18.0 for Windows (StataCorp. 2021. *Stata Statistical Software: Release 18*. College Station, TX: StataCorp LLC).

## Results

### Characteristics of the study population

A total of 75,250 women were new users of ALN or ZOL in the study period. Among men, we identified 12,739 new users of ALN and ZOL (Table 1). Women who were ever exposed to ZOL had a somewhat younger age at initiation than women ever exposed to ALN, with a mean of 67.8 versus 69.8 years, and they also had a slightly higher comorbidity level. Being married or in a registered partnership was slightly more common in women ever exposed to ZOL, and these women more often had higher education and lived in more urban areas. Also in men, ZOL users were on average younger than ALN users, but comorbidity level did not differ. Being highly educated and living in an urban area was slightly more common in men who were ever exposed to ZOL.

**Table 1:** Crude characteristics of the study population by use of alendronate and zoledronic acid with osteoporosis as indication, Norwegians 50 years and older in 2005–2016

	Women (75,250)		Men (12,739)	
	Users of alendronate	Users of zoledronic acid	Users of alendronate	Users of zoledronic acid
Individuals^a^ (%)	72,614 (96.5)	6,366 (8.5)	12,311 (96.6)	784 (6.2)
Years under observation, mean (SD)	4.6 (3.2)	4.4 (2.9)	3.9 (2.9)	3.8 (2.7)
Years under specific exposure, mean (SD)	3.1 (2.7)	2.4 (1.7)	2.6 (2.4)	2.3 (1.8)
Years under any exposure, mean (SD)	3.2 (2.7)	3.5 (2.4)	2.7 (2.4)	3.0 (2.3)
Age at first exposure^b^, mean (SD)	69.8 (9.7)	67.8 (8.9)	70.6 (9.9)	67.8 (10.2)
Rx-Risk score at first exposure^b^, median (interquartile range)	4 (0–9)	5 (1–10)	6 (2–11)	6 (2–11)
Ever-user of teriparatide, %	1.1	5.3	1.0	6.8
Ever-user of glucocorticoid, %	47.0	49.4	62.0	52.6
Individuals missing Rx-Risk Score^c^ at first exposure^b^, number (%)	940 (1.3)	50 (0.8)	167 (1.4)	12 (1.5)
Married or registered partner^d^, %	62.8	65.0	73.0	72.2
Education (in 2001), %				
Low	32.3	24.7	27.6	22.5
Medium	49.8	51.8	52.1	51.7
High	17.2	22.7	19.5	25.1
Missing	0.8	0.9	0.8	0.8
Urbanization^d^, %				
Rural	8.9	7.4	10.1	9.2
Suburban	23.2	17.8	24.7	20.7
Urban	67.9	74.8	65.3	70.2

^a^User groups are not mutually exclusive

^b^Referring to first exposure to the specific drug in each column

^c^Missing Rx-Risk score indicates no prescription drug use in previous years

^d^There were no missing values in any group

### Duration of treatment

Of the 75,250 exposed women in the study, 72,502 (96.3%) started with ALN, 2,707 (3.6%) started with ZOL, and 41 (0.1%) initiated ALN and ZOL concurrently. When considering use at any time during the study period, 6,366 (8.5%) women filled prescriptions for ZOL, 3,730 (5.0%) had exposure to both drugs, and 2,487 (3.3%) had concurrent exposure to both drugs. Among the women who used both drugs during the study period (3,730), almost all started with ALN (3,618 [97.0%]). These women had a median duration of ALN exposure of 13 months (interquartile range [IQR]: 7–30) before switching to ZOL. At the end of follow-up, 29,568 women (39.3%) were on neither treatment, whilst 4,496 women (6.0%) were treated with ZOL, and 41,186 women (54.7%) were treated with ALN. For women ever-exposed to ALN who discontinued treatment before end of follow-up, median total treatment was 22 months (IQR: 9–50). And for women ever-exposed to ZOL and who discontinued treatment, it was 38 months (IQR: 22–59). For women ever-exposed to ALN, the status at end of observation was sustained hip fracture for 5.3%, death for 15.1% and emigration for 0.1%; 79.5% were alive and still hip fracture free. Equivalently, for women ever-exposed to ZOL, 2.4% sustained a hip fracture, 5.7% died, 0.1% were emigrated, and 91.8% were alive and still hip fracture free at the end of their observation.

Of the 12,739 exposed men in the study, 12,292 (96.5%) started with ALN, 437 (3.4%) started with ZOL, and 10 (0.1%) filled prescriptions for both drugs concurrently. A total of 784 (6.2%) men used ZOL at any time during the observation period, 356 (2.8%) had exposure to both drugs, and 253 (2.0%) had concurrent exposure to both drugs at some point in time. Almost all men who used both drugs during the study period started with ALN (337 [94.7%]). These men had a median duration of ALN exposure of 12 months (IQR: 7–27) before switching to ZOL. At the end of follow-up, 4,858 men (38.1%) were on neither treatment, whilst 559 men (4.4%) were treated with ZOL, and 7,322 men (57.5%) were treated with ALN. Median total treatment time for the men who discontinued treatment before end of follow-up was 17 months (IQR: 9–36) for men ever exposed to ALN and 31 months (IQR: 18–49) for men ever exposed to ZOL. For men ever-exposed to ALN, the status at end of observation was sustained hip fracture for 4.7%, death for 28.8% and emigration for 0.1%; 66.4% were alive and still hip fracture free. Equivalently, for men ever-exposed to ZOL, 1.9% sustained a hip fracture, 12.9% died, none were emigrated, and 85.2% were alive and still hip fracture free at the end of their observation.

### Fracture prevention

2,193 hip fractures occurred in women on current ALN treatment (incidence rate 9.8 per 1000 person-years), and 96 occurred in women on current ZOL treatment (incidence rate 6.9 per 1000 person-years) (Table 2). The corresponding numbers for men were 365 (11.5) and 11 (6.8) (Table 3).

**Table 2:** Hazard ratios (HRs) with 95% confidence intervals (CIs)^a^ for first hip fracture according to time-dependent use of zoledronic acid^b^ or discontinued treatment compared with alendronate in women aged 50 years and older in Norway 2005*–*2016. *N* = 75,250

			*Base model*			*Model I*^*c*^			*Model II*^*d*^		
Exposure	Person-time (years)	Hip fractures	HR	*95% CI*	*p-value*	HR	*95% CI*	*p-value*	HR	*95% CI*	*p-value*
Alendronate	223,923	2193	1.00 (ref)	*–*	*–*	1.00 (ref)	*–*	*–*	1.00 (ref)	*–*	*–*
Zoledronic acid	13,890^e^	102	0.90	*0.73–1.09*	*0.28*	0.85	*0.69–1.03*	*0.10*	0.75	*0.61–0.91*	*0.004*
Discontinued treatment	99,987	1610	1.43	*1.35–1.53*	< *0.001*	1.42	*1.33–1.51*	< *0.001*	1.33	*1.24–1.42*	< *0.001*

^a^Flexible parametric survival model with age (months) as timescale

^b^With osteoporosis as indication

^c^Adjusted for Rx-Risk score, ten-year birth cohort, marital status (married/other), education level, and level of urbanization of place of residence

^d^Adjusted for the same as model I and in addition adjusted for accumulated alendronate exposure

^e^8.4% of this person-time was double-exposed time with both zoledronic acid and alendronate

**Table 3:** Hazard ratios (HRs) with 95% confidence intervals (CIs)^a^ for first hip fracture according to time-dependent use of zoledronic acid^b^ or discontinued treatment compared with alendronate in men aged 50 years and older in Norway 2005*–*2016. *N* = 12,739

			*Base model*			*Model I*^*c*^				*Model II*^*d*^	
Exposure	Person-time (years)	Hip fractures	HR	*95% CI*	*p-value*	HR	*95% CI*	*p-value*	HR	*95% CI*	*p-value*
Alendronate	31,712	365	1.00 (ref)	*–*	*–*	1.00 (ref)	*–*	*–*	1.00 (ref)	*–*	*–*
Zoledronic acid	1772^e^	11	0.66	*0.36–1.21*	*0.18*	0.70	*0.38–1.27*	*0.24*	0.59	*0.32–1.07*	*0.08*
Discontinued treatment	15,257	214	1.15	*0.97–1.37*	*0.10*	1.24	*1.04–1.47*	*0.015*	1.13	*0.95–1.35*	*0.17*

^a^Flexible parametric survival model with age (months) as timescale

^b^With osteoporosis as indication

^c^Adjusted for Rx-Risk score, ten-year birth cohort, marital status (married/other), education level, and level of urbanization of place of residence

^d^Adjusted for the same as model I and in addition adjusted for accumulated alendronate exposure

^e^8.3% of this person-time was double-exposed time with both zoledronic acid and alendronate

For women, when including full adjustment, current ZOL exposure was associated with a lower hazard ratio for hip fracture, compared to current ALN: HR 0.75 (95% CI: 0.61–0.91). There was also a statistically significant *increase* in risk of hip fracture after discontinued use.

For men, current ZOL vs. ALN treatment gave a HR of 0.59 (95% CI: 0.32–1.07, Table 3). Transitioning from either treatment to no treatment incurred a higher risk than current ALN treatment in Model 1: HR 1.24 (95% CI: 1.04–1.47), but this result did not remain statistically significant after adjustment for accumulated ALN exposure.

### Time-varying estimates

**Fig. 1:**
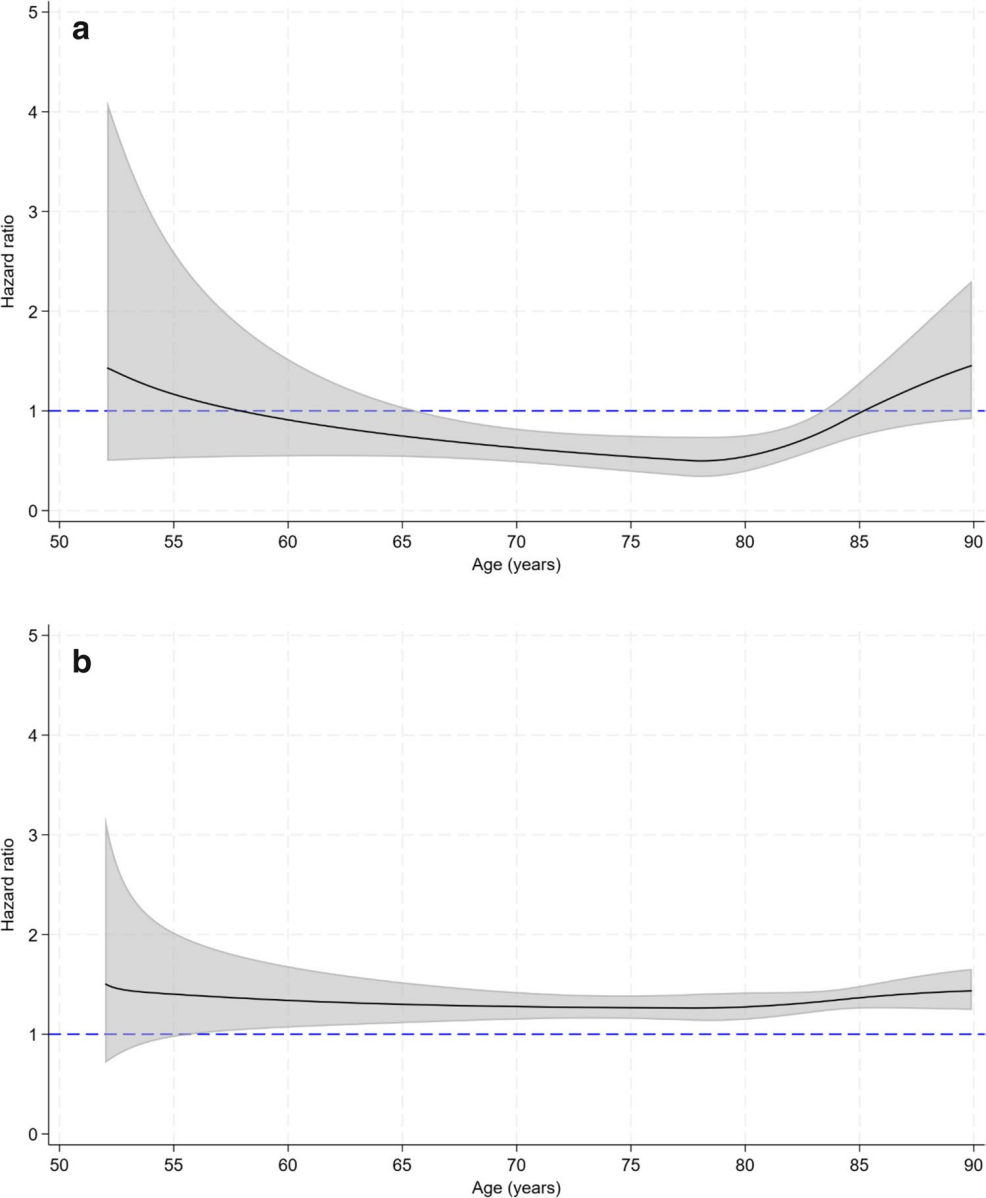
**a** Time-dependent hazard ratios with 95% confidence intervals for hip fracture according to use of ZOL compared with ALN in women in Norway, ages 52–89. Adjusted for marital status, ten-year birth cohort, education level, urbanization of place of residence and accumulated exposure to ALN (Model II). **b** Time-dependent hazard ratios with 95% confidence intervals for hip fracture according to discontinued use of ZOL or ALN compared with current ALN use in women in Norway, ages 52–89. Adjusted for marital status, ten-year birth cohort, education level, urbanization of place of residence, and accumulated exposure to ALN (Model II)

The HR of hip fracture in women treated with ZOL compared to women treated with ALN varied across age (Fig. 1a). The risk of hip fracture was significantly lower in women using ZOL between the ages of approximately 66 and 83 years. The risk of hip fracture associated with discontinued use was higher at all ages and statistically significantly so from the age of approximately 56 years (Fig. 1b).

**Fig. 2:**
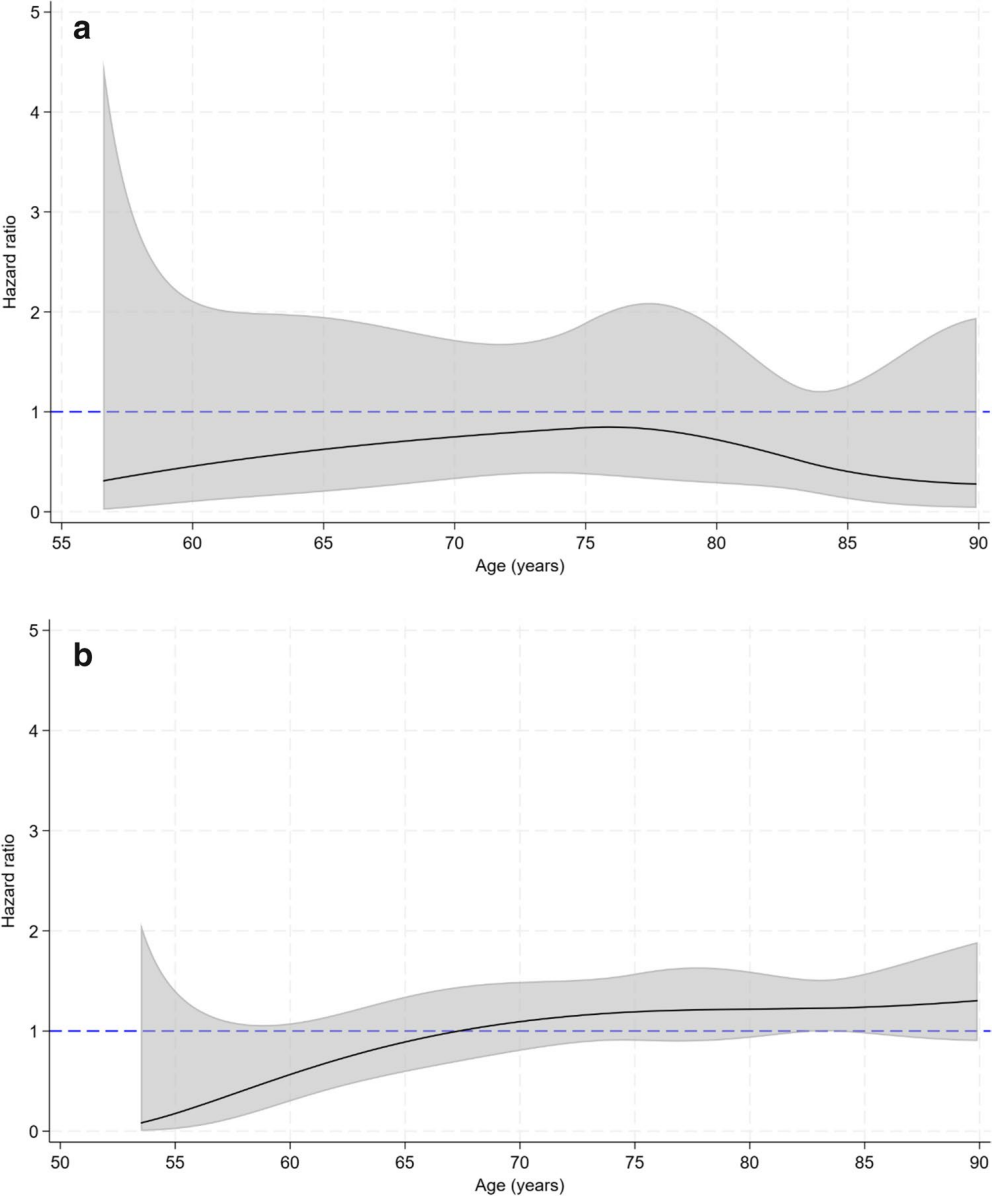
**a** Time-dependent hazard ratios with 95% confidence intervals for hip fracture according to use of ZOL compared with ALN in men in Norway, ages 57–89. Adjusted for marital status, ten-year birth cohort, education level, urbanization of place of residence, and accumulated exposure to ALN (Model II). **b** Time-dependent hazard ratios with 95% confidence intervals for hip fracture according to discontinued ZOL or ALN compared with current ALN use in men in Norway, ages 54–89. Adjusted for marital status, ten-year birth cohort, education level, urbanization of place of residence and accumulated exposure to ALN (Model II)

When analyzed across age, the point estimate for men was in favor of ZOL for all ages, but statistically non-significant at all ages (Fig. 2a). In the time-dependent analysis, the hip fracture risk associated with discontinued use did not differ significantly from the risk associated with current ALN use (Fig. 2b).

In the additional stratified analyses by treatment duration in women, the risk of hip fracture associated with current ZOL exposure was above that for ALN exposure at treatment durations shorter than one year. At treatment durations of one year or more, the trend was in favor of ZOL (Table 4).

**Table 4:** Hazard ratios (HRs) with 95% confidence intervals (CIs)^a^ for first hip fracture according to current use of zoledronic acid^b^ of different accumulated durations compared with current use of alendronate of the same accumulated duration, in women aged 50 years and older in Norway 2005*–*2016. *N* = 75,250

				*Model II*^*c*^		
Treatment duration	Drug	Person-time (years)	Hip fractures	HR	*95% CI*	*p-value*
1*–*5 months	Alendronate	19,253	341	ref	*–*	*–*
	Zoledronic acid	658	13	1.41	*0.81–2.46*	*0.22*
6*–*11 months	Alendronate	34,330	328	ref	*–*	*–*
	Zoledronic acid	2020	27	1.73	*1.17–2.56*	*0.006*
12*–*23 months	Alendronate	47,435	448	ref	*–*	*–*
	Zoledronic acid	4934	22	0.56	*0.37–0.87*	*0.009*
24*–*35 months	Alendronate	35,409	325	ref	*–*	*–*
	Zoledronic acid	3187	19	0.74	*0.46–1.17*	*0.20*
36 months or more	Alendronate	87,495	751	ref	*–*	*–*
	Zoledronic acid	4360	21	0.59	*0.38–0.92*	*0.020*

^a^Flexible parametric survival model with age (months) as timescale

^b^With osteoporosis as indication

^c^Adjusted for Rx-Risk score, ten-year birth cohort, marital status (married/other), education level, level of urbanization of place of residence, and accumulated exposure to alendronate

### Sensitivity analyses

In a sensitivity analysis censoring women at age 85 years, the adjusted HR of hip fracture for ZOL use compared to ALN use was 0.66 (95% CI: 0.52–0.83). The adjusted HR of hip fracture when treatment was discontinued was 1.29 (95% CI: 1.20–1.40). If age was further restricted to younger than 80 years, adjusted HR was 0.57 (95% CI: 0.43–0.76) for current ZOL treatment compared with current ALN treatment, and 1.23 (95% CI: 1.11–1.36) for discontinued treatment compared with current ALN treatment. The proportions of ever-users of teriparatide during the study period (2005*–*2016) were 1.1% among women ever-exposed to ALN (*n*=818) and 5.3% among women ever-exposed to ZOL (*n*=334). In the sensitivity analysis adjusting for ever-use of teriparatide in women, the main results were largely unchanged; adjusted HR of hip fracture was 0.71 (95% CI: 0.58–0.88) for current ZOL vs. ALN exposure, and 1.33 (95% CI: 1.24–1.41) for discontinued treatment vs. current ALN exposure. The proportions of ever-users of glucocorticoids, sedatives/anxiolytics and opioids were very similar between the two treatment statuses, and separate sensitivity analyses including adjustment for each of these drug classes produced similar results to those of the main adjusted analysis.

## Discussion

This nationwide study included 75,250 women and 12,739 men aged 50–89 years who were treated with ALN or ZOL for osteoporosis in outpatient care. Current treatment with ZOL was associated with a 25% lower risk of hip fracture for women compared with ALN treatment (HR: 0.75; 95% CI: 0.61–0.91). The same trend was apparent among men, but with statistically not-significant estimates. In women, the lower risk of hip fracture when treated with ZOL was apparent in the age range 66–83 years. Both women and men had increased risk of hip fracture if treatment was discontinued.

Imminent fracture risk is expected to be very high when initiating bisphosphonates. Our sensitivity analysis in women showed a greater risk during the first year of ZOL treatment compared to the first year of ALN. In the following period (12–23 months), the risk was significantly lower in women using ZOL compared to women using ALN. This duration will typically correspond to having received a second infusion of ZOL. The risk estimates for treatment durations of two years or longer continued to be in favor of ZOL. The higher risk seen in the early phases of ZOL treatment might represent a difference in patients’ inherent risk; high-risk patients might be more frequently treated with ZOL than with ALN. For women, the comorbidity level was slightly higher when initiating ZOL, also supporting a higher initial fracture risk.

In our study, there is information on prescriptions filled, and not drugs actually administered. As ZOL is a yearly infusion, and ALN is a weekly pill, better adherence to treatment may be one factor behind the apparent better effectiveness of ZOL.

The increase in risk of hip fracture seen for both sexes after treatment discontinuation is of clinical relevance. Our results support that when treatment for some reason is withdrawn, fracture risk increases. As patients were censored if they switched to other types of anti-osteoporosis drugs, those who transitioned to an unexposed status were likely correctly classified as currently untreated. A possible exception is if individuals were institutionalized and received continued anti-osteoporosis treatment in institution, as only drugs dispensed in outpatient pharmacies were recorded. By the end of 2016, 1.2% of the Norwegian population aged 50–89 years were permanent nursing home residents (www.ssb.no/en). In the sensitivity analyses restricting highest attained age to 85 and 80 years, respectively, use of ZOL was associated with an even lower risk, and the increased risk of hip fracture for discontinued treatment compared to ALN treatment persisted. The proportion of long-term institutionalized women increases with age (www.ssb.no/en), and so will the risk of misclassifying women in terms of (apparent) discontinued exposure. Hence, by limiting the statistical analysis to younger age groups, this problem diminished, and we observed that the results remained similar and statistically significant, supporting the results of the main analysis. Patients who discontinue use might also have a greater fracture risk than those who persist with treatment for a variety of reasons connected to healthcare behavior or other clinical reasons for discontinuation. We expect that some of the differences in the healthcare seeking behavior were accounted for through the included variables of educational level and marital status.

As the median total treatment time among women ever exposed to ALN who discontinued treatment before the end of follow-up was 1.8 years, many did not reach the recommended treatment duration of five years, after which a “drug holiday” is normally considered [28, 29, 35, 36]. Median total treatment time for women ever exposed to ZOL who discontinued treatment before end of follow-up was 3.2 years. The observed increase in hip fracture risk after discontinued treatment, combined with the well-described undertreatment of osteoporosis in Norway and in Europe [3, 37], implies an unrealized potential for pharmacological fracture prevention. The results also imply that clinicians should have a low threshold for choosing ZOL over ALN for patients at risk of hip fracture.

### Strengths and limitations

Strengths of this study included use of individual-level data from registries with objective recording of drug dispensing and hip fractures over 12 years. Owing to Norway’s universal public healthcare coverage and each resident’s unique personal identification number, our registry data cover the population in Norway. This study had a prevalent new-user design with an adjusted direct comparison of the hazard rates of hip fracture for two different drug exposures [24]. This design and statistical analysis allowed for utilizing the longitudinal data available in the NorPD. Another important strength was the ability to adjust for the Rx-Risk score, a time-updated broad score for health, which we have recently shown to be strongly associated with risk of hip fracture [38].

There were also some limitations to our study. As it was based on a registry linkage, we lacked data on individual lifestyle characteristics which could differ across the compared treatments, such as, e.g., smoking and body mass index (BMI). These two factors have been shown to impact hip fracture risk in opposite directions, with smoking *elevating* the risk and higher BMI *lowering* the risk [39–42]. A higher smoking prevalence and a higher BMI are linked to a lower socioeconomic status [43, 44]. Educational level and urban/rural dwelling varied slightly between treatments, and we expect to have accounted for some unmeasured confounding by adjusting for these covariates.

The NorPD includes filled prescriptions in outpatient pharmacies only and does not cover drugs given in institutions. As some patients receive ZOL in hospitals and nursing homes and were thus not included, the number of ZOL initiators was limited despite the large sample size. Some of these patients will also have had a hip fracture recently, and thus not be eligible for the study. This affected statistical power and resulted in relatively wide CIs. It may also have introduced inaccuracy in the washout for previous ZOL use, as we cannot exclude that some individuals who received treatment with ZOL in a hospital setting in the 18 months prior to inclusion might have been incorrectly included as new users. Furthermore, this limitation might also have affected the validity of our estimates for discontinued use since it is possible that some individuals who were classified as currently unexposed might have received treatment at an institution circumventing a prescription filling at an outpatient pharmacy.

Individuals initiated on ALN and ZOL may have had different baseline risks of fragility fracture. An important risk factor for future hip fracture is a previous fragility fracture [45]. High-quality data on fragility fractures are hard to come by, also in Norway. Many fractures are not diagnosed, and many are diagnosed and treated on a primary level of care. This study is limited by its lack of data on previous fractures in the participants.

## Conclusion

We aimed to investigate whether zoledronic acid (ZOL) was associated with a lower risk of first hip fracture than alendronate (ALN) on a population level in Norway. The risk of hip fracture in women when treated with ZOL was 25% lower than when treated with ALN. Discontinued treatment was associated with a 33% increased risk of hip fracture relative to continued ALN treatment. The estimated HRs of hip fracture in men were similar to those found in women, albeit not statistically significant. Within the constraints of the study’s limitations, these findings show that ZOL is associated with a somewhat lower risk of hip fracture than ALN on a population level in a real-world setting, and support opting for ZOL when feasible for patients at risk of hip fracture. Similar studies should be performed on updated population data in the near future to verify these findings.

## Data Availability

Due to protection of privacy under the General Data Protection Regulation and Norwegian law, the individual-level data can only be made available after approval by the Regional Committee for Medical and Health Research Ethics and application to the respective data owners (Statistics Norway, the Norwegian Patient Registry, and the Norwegian Prescribed Drug Registry).

## Declarations

### Disclaimer

Data from the Norwegian Patient Registry have been used in this publication. The interpretation and reporting of these data are the sole responsibility of the authors, and no endorsement by the Norwegian Directorate of Health is intended nor should be inferred.
